# Cardiorespiratory and Neuroprotective Effects of Caffeine in Neonate Animal Models

**DOI:** 10.3390/ani13111769

**Published:** 2023-05-26

**Authors:** Daniel Mota-Rojas, Dina Villanueva-García, Ismael Hernández-Ávalos, Alejandro Casas-Alvarado, Adriana Domínguez-Oliva, Karina Lezama-García, Agatha Miranda-Cortés, Julio Martínez-Burnes

**Affiliations:** 1 Neurophysiology, Behavior and Animal Welfare Assessment, Universidad Autónoma Metropolitana (UAM), Mexico City 04960, Mexico; 2 Division of Neonatology, National Institute of Health, Hospital Infantil de México Federico Gómez, Mexico City 06720, Mexico; dinavg21@yahoo.com; 3 Clinical Pharmacology and Veterinary Anesthesia, Facultad de Estudios Superiores Cuautitlán, Universidad Nacional Autónoma de México (UNAM), Cuautitlán 54714, Mexico; mvziha@hotmail.com (I.H.-Á.);; 4 Facultad de Medicina Veterinaria y Zootecnia, Universidad Autónoma de Tamaulipas, Victoria City 87000, Mexico

**Keywords:** positive inotropic effect, hypoxia, newborn, methylxanthine

## Abstract

**Simple Summary:**

Caffeine is a stimulant used in humans and animals to improve newborns’ respiratory and neurological responses. The use of caffeine after birth could increase neonate survival. However, due to the immature systems of animals at birth, caffeine use can have different results. This review aims to understand caffeine’s effects on respiratory and neurological systems in neonate animal models (rat and mouse pups, goat kids, lambs, and piglets).

**Abstract:**

Caffeine is widely used to improve neonatal health in animals with low vitality. Due to its pharmacokinetics and pharmacodynamics, caffeine stimulates the cardiorespiratory system by antagonism of adenosine receptors and alteration in Ca^+2^ ion channel activity. Moreover, the availability of intracellular Ca^+2^ also has positive inotropic effects by increasing heart contractibility and by having a possible positive effect on neonate vitality. Nonetheless, since neonatal enzymatic and tissular systems are immature at birth, there is a controversy about whether caffeine is an effective therapy for newborns. This review aims to analyze the basic concepts of caffeine in neonatal animal models (rat and mouse pups, goat kids, lambs, and piglets), and it will discuss the neuroprotective effect and its physiological actions in reducing apnea in newborns.

## 1. Introduction

Caffeine or 1,3,7 tri-methylxanthine was first introduced to manage prematurity apnea (AOP) at the McGill University Hospitals in the mid-1970s to produce pharmacologic respirogenesis and reduce the need for intubation and mechanical ventilation in preterm neonates with recurrent AOP. Caffeine has a similar structure to adenosine [1]. Caffeine crosses all biological membranes and distributes into all body fluids [2]. When administered orally, it reaches up to 90% absorption [1].

It is a stimulant of the cerebral cortex and thus modifies the activity of the Central Nervous System (CNS) [3]. Adenosine receptors are present in different tissues, such as the CNS, lung, heart, and skeletal muscle, where caffeine has a stimulating effect. For example, caffeine improves cognitive ability due to the facilitation of synaptic capacity [4]. Caffeine enhances lung performance and capacity in the cardiorespiratory system by increasing both the air volume in each respiratory cycle and the cardiac contractility (positive inotropic effects) [5,6].

The physiological effects of caffeine have also been studied in neonates, where it has been suggested for the treatment of apnea, in addition to having a positive inotropic stimulus in animals with low vitality [7,8]. Studies regarding the effects of caffeine on the neurodevelopment of premature infants have been related to explain their poorly developed respiratory systems [9]. However, there is a need for more clarity about whether these benefits can persist due to the immaturity in the enzymatic and tissular systems that modify the activity of caffeine, possibly reducing its therapeutic efficacy. This review aims to analyze caffeine’s pharmacokinetic and pharmacodynamic characteristics in neonatal animal models (rat and mouse pups, goat kids, lambs, and piglets). It will discuss the neuroprotective effect and its physiological actions in reducing apnea in newborns.

## 2. Pharmacokinetic Characteristics of Caffeine in Neonate Animals

Caffeine is one of the most recognized plant-derived alkaloids for its stimulating effect on the CNS [10]. This methylxanthine is used in neonatology due to its pharmacokinetic characteristics in newborns since its oral bioavailability reaches 99% after 45 min of administration [11]. Given its absorption level, neonates present plasmatic concentrations between 5–20 mg/mL at a dose of 5–10 mg/kg/day [12]. From a comparative perspective, Menozzi et al. [13] studied in sows that the plasma concentration of caffeine was 13.77 ± 0.97 μg/ mL with a maximum concentration (Cmax) of 20.02 ± 1.51 μg/ mL at 9.51 h after 25 mg/kg orally. This would indicate that caffeine has a lower concentration in neonates due to its larger volume of distribution (Vd), where factors such as postnatal age and birth weight can influence its absorption [14].

Caffeine can readily diffuse into cellular tissues because it has a high Vd in infants (0.8–0.9 L/kg) [14]. According to Noh et al. [15], studies in rats report similar parameters with a Vd of 0.5 ± 0.1 L/kg. These data suggest that caffeine can have a high availability in the tissues and permeation in critical regions. These characteristics also allow caffeine to cross the blood-brain and placental barriers [16]. In this regard, a study on pregnant rats reported that caffeine crosses the placental barrier by passive diffusion, allowing a wide distribution in fetal tissues [17]. 

This methylxanthine is mainly biotransformed in the liver by the CYP1A2 enzyme, where three active metabolites, N-3 paraxanthine, N-7 theophylline, and N-1 theobromine have been identified in adult humans [18]. However, in infants, the main metabolite produced by demethylation is N-7 theophylline due to the immaturity of the main metabolic pathway [19]. Bienvenu et al. [20] evaluated the hepatic capacity to metabolize caffeine of neonate rats at different ages. The authors found that 25% of the caffeine metabolites at day 1 was N-1 theobromine, while this was 40% for older ages (seven days of age). This suggests that metabolism differs due to the possible immaturity of the enzymatic systems in the neonate. It is important that the hepatic metabolism does not accumulate this substance in excess, especially in the cases of dose dependent toxicities and hypersensitivity reactions [18].

The immaturity of the enzymatic systems in the newborn has a greater impact on the clearance rate of caffeine since it is slower at birth and increases with age as glomerular filtration increases [21]. According to the data in rats, the elimination rate of caffeine is 0.0109 L, with a half-life of 4.19 h in adult animals [22]. Although it is still not clear if this speed and elimination time may be higher in the newborn, it is known that 75% of caffeine is eliminated unchanged in the urine at this age [23].

Therefore, the evidence shows that caffeine in newborns has different pharmacokinetics due to circulatory immaturity. Moreover, the immaturity of the enzymatic systems and the limited renal perfusion can affect this drug’s elimination rate. Nonetheless, its physicochemical characteristics promote its distribution and permeation to most tissues, making it a pharmacological alternative for newborns.

## 3. Caffeine Pharmacodynamics in Neonates

Caffeine has a homologous molecular conformation to adenosine, making it a neuromodulator dependent on adenosine triphosphate (ATP). Its main mechanism is the non-selective antagonism of adenosine (A1, A2a, A2b, and A3) receptors, which are found predominantly in the CNS [24]. Immunohistochemical studies support that A1 and A2a receptors are found in high concentrations in the brain, with A1 receptors being ubiquitous in the region of the hippocampus and neocortex. In contrast, the A2a receptors are identified mainly in the striatum [25,26,27]. Adenosine is a neurotransmitter with diverse physiological functions, including the control of arousal, sleep, and cerebrovascular homeostasis. It has four known receptors: A1R, A2aR, A2bR, and A3R. Adenosine binding to its receptors leads to the inhibition of inspiratory neurons, resulting in central respiratory depression. Caffeine can non-specifically block these receptors, thereby indirectly stimulating the respiratory center, increasing sensitivity to carbon dioxide, enhancing diaphragm contractility, and improving the respiratory rate and tidal volume [28].

Antagonism of adenosine receptors decreases the activity of the phosphodiesterase enzyme, increasing adenosine 3′,5′-monophosphate cyclase (cAMP) [29]. This substance is an important mediator of second messenger signaling and modulates neurotransmitters. Therefore, it is related to the release of neurotransmitters, such as gamma-aminobutyric acid (GABA), norepinephrine, dopamine, serotonin, acetylcholine, and glutamate. This property influences its mechanism of action on brain adenosine receptors that modulate central noradrenergic, dopaminergic, serotonergic, cholinergic, GABAergic, and glutaminergic systems, which affects neuronal functioning. This facilitates neuronal transmission and higher cognitive performance due to greater chemical synapsing [30]. 

The decrease in cAMP alters neuronal modulation due to the adenosine inhibition signals on the neurotransmitters [31,32]. However, due to the location of A2a and A2b receptors in the lungs and heart, there is a controversy over whether this would be the primary mechanism of caffeine [33]. It has been described that activating the inhibitory G protein facilitates the mobilization of intracellular Ca^+2^ from the endoplasmic reticulum through the activation of ryanodine channels, which can be considered as a second mechanism of action [34]. Previous studies have described that caffeine reduces the activation threshold of ryanodine channels, facilitating the mobilization of intracellular Ca^+2^ to facilitate sympathetic neuron activity [35,36]. According to Kong et al. [37], caffeine markedly reduced the activation threshold of Ca^+2^ channels at the luminal level in cardiac myocytes. However, it did not affect the action threshold on the activation at the cytosolic level. This suggests that ryanodine channels modify the activation threshold as well as the cellular activity whose mechanism of action was initially suggested. 

The modification in the flow of Ca^+2^ affects skeletal muscle, since the increase of this substrate at the intracellular level in the myocyte could improve physical performance [38,39]. This was investigated by Sarbjit-Singh et al. [40], who evaluated the modulating effect of ryanodine on the activation of Na^+^ current in skeletal muscle fibers of the murine model. These authors mention that the activation and inactivation of the current-voltage by adding 0.5 and 2 mM of caffeine generated negative changes in the voltage dependence of the Nav 1.4 voltage-gated sodium channel and generated the gradual inactivation of ryanodine receptors that allowed the increase of Ca^+2^ ions at the cytosolic level. This could reaffirm that the negative modulation of the ion channels would positively modify the activity.

In summary, caffeine’s mechanism of action is in the antagonism of adenosine, altering the level of intracellular metabolites such as cAMP, which would affect the regulation of neurotransmitters in the neuronal synapse. The increased availability of neurotransmitters could facilitate neural activity. On the other hand, caffeine also alters the activity of Ca^+2^ ion channels, increasing the cytosolic levels of this ion and stimulating muscle activity (an ergonomic effect).

## 4. Stimulating Effect on the Respiratory Tract of the Newborn

Caffeine is the most commonly used drug in the neonatal intensive care unit (NICU) after antibiotics [8,41]. In recent years, an increasing number of high-quality clinical studies have demonstrated the protective effects of caffeine on the respiratory and nervous systems of premature infants. In this sense, it is argued that four biochemical mechanisms stimulate the respiratory function: (a) the mobilization of intracellular Ca^+2^, (b) the inhibition of phosphodiesterases, (c) the modulation of GABA_A_ receptors, and (d) the antagonism of A3 receptors [42]. These mechanisms modify the pulmonary response to hypoxia or the sensitization of the chemoreceptors to O_2_ and CO_2_ molecules [43]. It is also suggested that the stimulant effects of caffeine increase the sensitivity to CO_2_ in the respiratory centers, modifying the respiratory pattern [44]. 

Due to the chemoreceptor’s sensitization, it can reduce the periods of apnea in the newborn, thus improving its ventilatory dynamics. For example, in a pilot study in newborn baboons, Yoder et al. [45] found an association with an enhanced pulmonary mechanical function during the first 24 h of life when administering caffeine. Nevertheless, Crossley et al. [46] evaluated the effects of this drug on kidney and lung function in lambs at 126 days of gestation. These authors reported two main findings; that the administration of caffeine at 40 mg/kg had little effect on lung function —with no differences in PaO_2_ and hemoglobin oxygen saturation— and that the dose increased PaCO_2_ and pulmonary vascular resistance. 

The existing controversy questions the mechanism of action of caffeine when administered to newborns. In this sense, caffeine may improve pulmonary vagal afferents due to increased neural activity. A study of neonatal rabbits anesthetized with a barbiturate and receiving caffeine at 10 mg/kg reported an increased respiratory rate, minute volume, and respiratory flow, but caffeine did not improve vagal activity. These changes persisted in animals undergoing vagotomy [47]. 

The results shown above conclude that the improvement in neuronal synapsing caused by caffeine does not influence lung dynamics. It could possibly improve lung compliance, which would have a greater impact on the newborn’s tidal volume and lung capacity. It should be added that, according to what was indicated by Julien et al. [48], exposure to intermittent hypoxic states in rat pups given a 20 mg/kg dose of caffeine may result in an increase in minute volume, which was negatively correlated with apnea frequency in these animals (r^2^ = 0.52, *p* < 0.01). These results suggest caffeine affects apnea by increasing the central normoxic respiratory drive rather than a hypoxic response. On the other hand, it has been reported that 15 mg/kg of caffeine administered to rat pups had a 22% increase in the response to ventilation and a 15% increase in tidal volume, which led to the suggestion that caffeine possibly modifies the A1 adenosine receptor density [49]. 

Thus, caffeine modifies and optimizes respiratory control in the newborn due to an improvement in the disposition of adenosine receptors, as reported by Montandon et al. [50]. These authors studied the effect of caffeine on adenosinergic modulation through an antagonist of the A1 and A2 adenosine receptors in rats. The administration of caffeine and the A1 antagonist increased ventilation by 27%, reducing spontaneous apnea frequency with the administration of the A1 antagonist. In contrast, the A2 antagonist did not affect the ventilatory response. These findings support the theory that caffeine modifies the density of A1 receptors, improving ventilatory dynamics. The same authors reported that, in the carotid bodies of newborn rats receiving caffeine, it was possible to increase the mRNA expression of A2 and dopamine 2 receptors, improving the sensitization of respiratory chemoreceptors to hypoxia events [51]. Given the evidence, caffeine improves ventilatory dynamics by sensitizing chemoreceptors that can increase the ventilated tidal volume.

**Figure 1:**
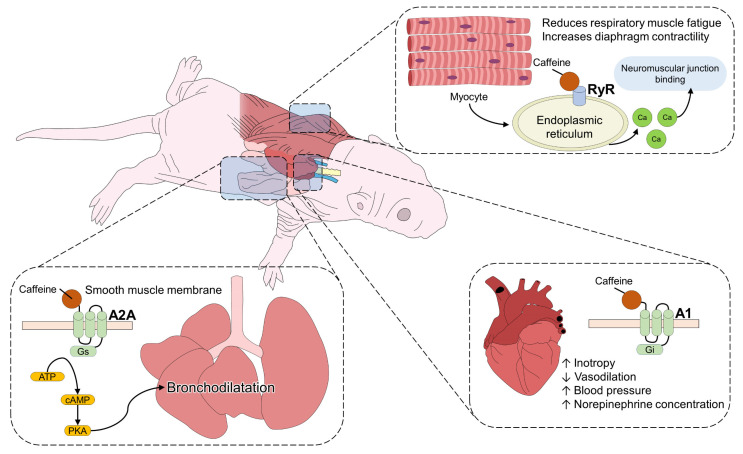
Cardiorespiratory effects of caffeine. Caffeine administration can act at three different levels to improve cardiorespiratory functions. At the skeletal muscle level, it directly induces the activation of RyR located in the muscle cell. This increases Ca^2+^ influx, which facilitates muscle contraction in the neuromuscular junction, reducing respiratory muscle fatigue and increasing diaphragm contractility and minute volume. In the lungs, caffeine blockade of A2A receptors causes bronchodilatation. In the heart, its inotropic effects cause tachycardia and blood pressure to rise. These effects of caffeine are beneficial in cases of neonatal apnea and hypoxia A2A: adenosine receptor 2A; AMP: adenosine monophosphate; cAMP: cyclic adenosine monophosphate; RyR: ryanodine receptors.

These studies suggest that caffeine increases the air volume that enters the neonate’s respiratory tract. From a clinical perspective, this strategy could facilitate the extubation of patients [52]. In addition, some authors have shown that caffeine also participates in the prevention and treatment of neonatal apnea episodes [53,54] (Figure 1). However, whether this benefit may be exclusive to caffeine or any of its metabolites has been questioned. Skouroliakou et al. [55] observed that standardized doses of caffeine and theophylline, in neonates with younger than 33 weeks of gestation, reduce apnea events significantly. In contrast, caffeine alone only controls apnea in newborns at risk. From these results, it can be inferred that caffeine alone helps to counteract apnea, but the derivatives of its metabolism contribute to the control of this event. Likewise, a meta-analysis focused on the effectiveness of caffeine (compared with amiodarone) as a treatment for apnea and was able to find that it would have similar effectiveness in infants but with the advantage of presenting fewer side effects [56]. Teng et al. [57] reported that hyperoxia exposure increased the expression of Bip, PERK, IRE1, sXBP1, cATF6, and CHOP during the cystic and alveolar stages of lung development, leading to lung injury due to oxidative stress and endoplasmic reticulum (ER) stress. Caffeine is proposed to reverse this oxidative damage, reduce apoptosis, and promote angiogenesis and alveolar development.

Bronchopulmonary dysplasia (BPD) is a chronic respiratory complication that affects a newborn’s early life. Three major postnatal pathological factors for BPD are the high-concentration of oxygen inhalation, the inflammatory response, and the mechanical ventilation. Although there have been attempts to adopt therapeutic protocols for this disease in clinical trials, there has been no significant decline in the incidence of BPD and some consequences have been reported with current therapies [58,59]. Lung injury caused by a high concentration of oxygen and mechanical ventilation results in the destruction of the alveolar structure, increased vascular permeability and an inflammatory response as some of the complications [60]. Caffeine has been recognized as a treatment for primary apnea in premature infants, and as a drug to prevent BPD in premature infants. When using caffeine in a placebo-controlled trial with preterm infants, the drug reduced the risk of BPD and inhibited the inflammatory response induced by hyperoxia exposure in infant rats [61]. The potential mechanism behind this reaction is the caffeine’s property to prevent lung tissular injury, reduce barotrauma, and improve ventilation and lung compliance. Contrarily, Dayanim et al. [62] showed that caffeine treatment exacerbated hyperoxic lung injury in neonatal rats, an important risk factor of BPD, which is another disorder that increases alveolar cell apoptosis. Caffeine may improve the prognosis of BPD by antagonizing the effect of prostaglandins. Nonetheless, to date, no therapeutic efficacy has been reported in animals (e.g., sheep fetuses) [63], and the timing, dosage, and side effects of caffeine use needs to be further examined.

Therefore, caffeine primarily induces sensitization of the chemoreceptors promoting an increase in the tidal volume of each respiratory cycle. Furthermore, this effect is related to the increased A1 and A2 receptors in the carotid bodies. Thus, these mechanisms can decrease the frequency of apnea and reduce ventilatory support.

## 5. Positive Inotropic Effect of Caffeine

Methylxanthines have a positive inotropic effect on the heart. Caffeine has also been reported to increase catecholamines and renin, both by peripheral and central effects. Some of the physiological responses are tachycardia, palpitations, rapid hypertension and a small decrease in heart rate in adults [64]. There is controversy on the arrhythmogenic potential of caffeine ingestion; however, the results are inconclusive and this has been proved only in animals and in humans with preexisting premature ventricular beats [6]. In general, methylxanthines have a positive inotropic effect on the heart. Based on a meta-analysis, caffeine at a dose of 400 mg affects the cardiac conduction system, increasing its frequency [6,10,65]. Moreover, this effect could be attributed to the alteration in the flow of Ca^+2^ due to the modification of the activity in the adenosine receptors [66]. This effect was evaluated by Rasmussen et al. [67] in cell cultures of ventricular myocytes of chick embryo. In this study, caffeine caused a 5–12% increase in the contraction amplitude and a 10 mV decrease in the membrane diastolic voltage. These events occurred with the increased Ca^+2^ release from the sarcoplasmic reticulum. Therefore, caffeine has a positive effect on cardiac contractility due to the activity of ryanodine channels. 

In neonates, this mechanism could be altered by the immature cardiac tissue that has less Ca^+2^ dependence. In this regard, Miller et al. [68] observed that both mature and immature rabbit myocytes presented a strong rapid contractility response that did not depend on extracellular Ca^+2^ but on the reserves in the sarcoplasmic reticulum, which decreased as these reserves were depleted. The observations made by these authors complement the idea that caffeine induces changes in intracellular Ca^+2^, stimulating cardiac contractility in newborns [69].

This inotropic effect of caffeine may have a clinical application in animals with low vitality, as indicated by Villanueva-García et al. [70]. These authors suggest that caffeine can stimulate cardiac contractility in newborn animals and thus increase vitality, which could guarantee survival. In addition, this was the main objective in a study by Robertson et al. [71], where they evaluated caffeine’s effect on Merino lambs’ survival rate. In this study, caffeine treatment reduced daily and first-week mortality compared to a control treatment. The authors attributed this increase in vitality to the stimulation of cardiac contractility and the reduction in hypoxia events upon drug administration. Therefore, caffeine enhances heart contractions by increasing the availability of intracellular Ca^+2^. This way, it could increase vitality and survival in weak animals at birth. Although caffeine can be considered to have an advantage in inducing a positive inotropic effect, it is discussed that it may present disadvantages, such as the induction of arrhythmias or the increase in blood pressure that can affect microcirculation in peripheral tissues, as at the renal level, however this can be induced dose-dependently [72,73]. For this reason, it is necessary to consider the therapeutic dose, which may be different between adult animals and neonates, as shown in Table 1.

## 6. Caffeine in Neuroprotection

Using caffeine in mouse newborns has been a valuable strategy for reducing neonatal hypoxic-ischemic brain injury [81,82]. It has been used as a standard in all intensive care units (methyl theobromine) [83], replacing other treatments used in cases of apnea, such as theophylline and aminophylline [12]. In a murine model with germinal matrix-intraventricular hemorrhage (GM-IVH), a disorder associated with comorbidities such as cerebral palsy, sensory and motor impairment, learning disabilities, or neuropsychiatric disorders, Alves-Martinez et al. [84] analyzed two doses of caffeine (10 and 20 mg/kg) to treat this disorder. According to the results, both doses reduced hemorrhage burden. The drug showed a general neuroprotective effect in their model while diminishing brain atrophy and ventricle enlargement. 

In addition, the therapeutic cardiorespiratory effects of caffeine in the newborn might promote vitality. It has been reported that due to the abundant distribution of adenosine receptors, the neuroprotective effect of caffeine in newborns could be observed since apnea induces hypoxia and ischemia events in the brain, leading to neurotoxicity and degeneration of the white matter [85]. In infants, Schmidt et al. [86] reported that caffeine administration in newborns significantly decreased cerebral palsy, bronchopulmonary dysplasia, patent ductus arteriosus requiring medical and/or surgical treatment, and severe retinopathy of prematurity. However, just as protective and positive effects have been observed in human newborns, animal studies have shown conflicting results regarding caffeine’s role in neurodevelopment [87]. 

Cardiorespiratory effects have been reported at doses of 5–20 mg/kg, and neuroprotective effects have also been observed in newborns at this dose. For example, Winerdal et al. [81] conducted a randomized study in WT C57/bl6 rats in which a single dose of caffeine was administered at 5 mg/kg. Caffeine significantly reduced the presence of CD69+ and CD8 in the brain 24 h after treatment. In addition, there was a 44% decrease in the atrophy or damage to brain functions in the treated rats compared to the control group (treated with phosphate buffered saline). The authors concluded that its administration decreased brain atrophy and improved motor function in the open field test. Similarly, in a systematic review conducted by Bruschettini et al. [87], it was found that caffeine at doses of 5–20 mg/kg had a positive effect on the general functionality of the animals since they were able to observe a better performance in the maze tests carried out in the rats and mice studied.

Yang et al. [88] also reported neuroprotective properties in a neuronal proteomic analysis in newborn rats with induced hypoxia receiving caffeine. These authors found in the immunohistochemical analysis that the levels of myelin basic protein, proteolipid protein, myelin-associated glycoprotein precursor, and sirtiun 2 were reduced significantly with caffeine treatment in hypoxia-ischemic animals. Additionally, caffeine was found to enhance the expression of synaptophysin and postsynaptic density protein. These results demonstrate that caffeine has a neuroprotective effect by reducing the inflammatory process in the CNS. These data were reaffirmed in a subsequent study by these same authors. They evaluated Sprague-Dawley rats that were induced with cerebral hypoxia-ischemic ligation of the common carotid artery and were subsequently treated with caffeine. They observed that caffeine inhibited the activation of the NLRP3 inflammasome, negatively regulated the expression of the CD86 protein and iNOS, and inhibited the transcription of TNF-α and IL-1β, which would support the idea that caffeine reduces the inflammatory process and thus has a positive effect on the cognitive performance in the newborn [89].

**Figure 2:**
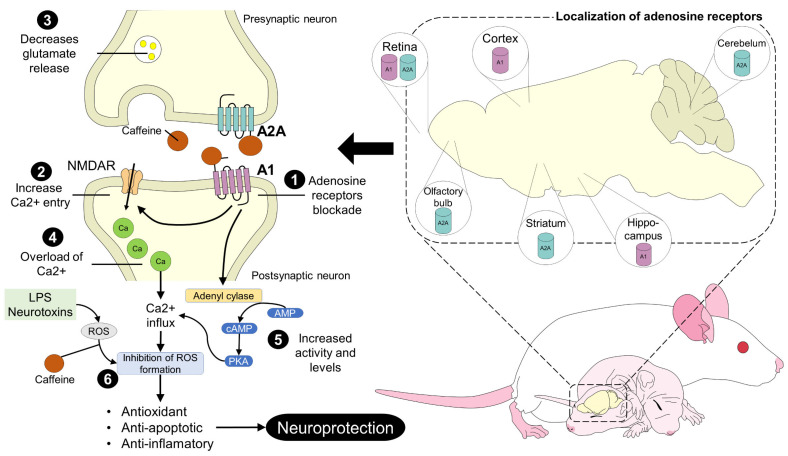
Neuroprotective properties of caffeine in the neonate’s brain. Caffeine interacts with adenosine receptors (A2A and A1) located in cerebral structures such as the striatum, cerebellum, and olfactory bulb, among others, by binding to these receptors. In the presynaptic and postsynaptic neurons, the blockade of A2A and A1 receptors, respectively, causes a series of changes to enhance the neuroprotective properties of caffeine. Binding to the receptors causes an increase in Ca^2+^ entry through NMDAR, and increases the activity of adenyl cyclase, cAMP, and PKA while decreasing glutamate release. This causes a Ca^2+^ overload and upregulation of factors that inhibit ROS formation, giving the antioxidant, antiapoptotic, and anti-inflammatory effects of caffeine. AMP: adenosine monophosphate; cAMP: cyclic adenosine monophosphate; LPS: lipopolysaccharides; PKA: protein kinase A; ROS: reactive oxygen species.

In this context, Sun et al. [90] evaluated the effect of brain activity and tissue neuroprotection in newborn rats from mothers treated with caffeine. They found that caffeine reduced brain injury by 1.6 ± 4.5% and likewise increased the duration and amplitude of activity on electroencephalography. These data again corroborate caffeine’s neuroprotective effect by reducing the inflammatory response at the brain level, and consequently, hypoxia. If the pharmacodynamic effects of caffeine are considered, it is possible to understand that adenosine receptor agonism helps in the expression of neurotransmitters and thus improves neuronal activity [70,82] (Figure 2).

Based on all of the above, it can be concluded that caffeine could be a therapy to mitigate the effects of neonatal hypoxic-ischemic brain injury. It may help decrease the burden of morbidities in preterm neonates [87]. 

## 7. Future Directions

Besides caffeine use in neonates, other factors, such as the ideal timing of administration and the interaction of caffeine with other drugs used in neonatology are relevant fields of research. Some studies have shown that pre-weaning mortality in piglets increases if caffeine at 30 mg is given orally at birth (*p* < 0.05), contrarily to doses at eight and 12 h post farrowing [91]. The use of caffeine with other drugs, their interaction, and the possible outcome in neonates are other topics that need to be considered. Caffeine and glucose supplementation to piglets at birth is a method used for reducing neonatal mortality and providing energy resources. However, this was only reported in low-birth-weight piglets administered with 30 mg of caffeine and 300 mg of glucose. Growth was improved in the first three days of life without affecting mortality, temperature, or colostrum intake [92]. Through Physiologic Based Pharmacokinetics (PBPK), and its combination with pharmacodynamics, the possible effects of caffeine could be determined to establish the desired and effective drug profile in preterm neonates (e.g., post-anesthesia/post-surgical apnea control, weaning from mechanical ventilation and extubating) or individualized medicine in newborns [2]. 

The maternal supplementation of caffeine is another approach suggested to improve newborn health. In Merino ewes, caffeine at 20 mg/kg resulted in lambs with higher rectal temperatures (*p* = 0.021), greater immunoglobulin concentrations (*p* = 0.041), and more suckling attempts than control animals and those receiving only 10 mg/kg [93]. A similar result was reported by Dearlove et al. [94] in piglets from sows receiving 2g of caffeine three days before farrowing. In this study, treated sows gave birth to fewer stillborn (*p* = 0.05), but no effect was reported on the viability score. In contrast, ewes receiving 10 mg/kg the day before lambing and those receiving 20 mg/kg in a four-week protocol did not improve lamb mortality and weight gain, concluding that caffeine is not an effective treatment to enhance perinatal survival in the species [95]. Caffeine is known to cross the placental barrier; however, little is known about the long-term impact of gestational caffeine exposure (GCE) on neurodevelopment. In the mouse brain, an alteration of neuron and neural circuits after GCE has been reported. Similarly, in children nine to ten years old, an Adolescent Brain and Cognitive Development^sm^ (ABCD ®) study performed by Christensen et al. [96] registered that GCE alters the developmental trajectory of white matter and neurocognition into adolescence. Nonetheless, further research towards this topic is necessary to validate its effects on neonatal neurocognition. The ambivalence in the results is why caffeine supplementation, not only in neonates, but also in the mother, is a topic that deserves future research.

Other topics that deserve to be fully studied are the exact mechanisms of action of hypothermia, genetics, circadian circle, and caffeine treatment. For hypothermia, it is thought that caffeine promotes energy preservation and reduces cytotoxic edema, free radicals, inflammation, and apoptotic cell death due to its anti-inflammatory properties. However, since factors like behavioral and neuroprotective outcomes might differ according to sex, this comparison needs to be considered to translate from animal to human [97]. On the other hand, in preterm infants with AOP, phenotypes and the expression of certain receptors have been associated with caffeine treatment and efficacy. Guo et al. [98] studied the circadian clock and its relation with the aryl hydrocarbon receptor (AHR) signaling pathways in preterm babies. From 104 individuals, the results showed that AHR genetic variations (rs1476080 and rs2066853), but not AHRR or ARNT genes, influence caffeine therapy’s efficacy. Regarding circadian rhythms in premature infants and AOP management, preterm infants experience ultradian or irregular rhythms during early postnatal life [99]. In kid goats, Piccione et al. [100] has reported that the maturity of the circadian rhythm does not mature until the end of the second year of life of the animals. Moreover, 40-day-old foals have blood pressure immaturity [101], a factor that need to be considered when trying to administer caffeine to neonates. Also, caffeine has been shown to alter circadian rhythms in humans and animals [99]. Novel treatment using caffeine could open a field where caffeine treatment could be coordinated with circadian rhythms to improve disease management and care for premature infants.

## 8. Conclusions

Caffeine and related methylxanthines, in humans and animals, cross all biological membranes and distribute in all body fluids, without accumulating in tissues and organs. Caffeine is essential in stimulating the CNS, lung, heart, and skeletal muscle, improving cognitive abilities, lung capacity, and cardiac contractility. The benefits of caffeine administration to neonates include the reduced risk of hypoxic ischemia, cerebral palsy, bronchopulmonary dysplasia, patent ductus arteriosus, and retinopathy. Moreover, it decreases inflammatory processes and could positively affect the newborn’s cognitive development. However, it is important to clarify that its dose, route, time of administration, and species must be considered to define whether its application may be favorable. 

Since caffeine has a high availability in tissues and permeation in critical regions, it can cross the blood-brain and placental barriers, still allowing this substance to be absorbed by the fetus. However, as it is metabolized in the liver and fetuses and newborns do not have these processes fully developed, only a quarter of caffeine is metabolized in the newborn due to the immaturity of the enzymatic systems.

Although it is a substance that has been widely used in human and non-human newborns, there is still much research to be done to define its benefits and adverse effects in different species of domestic animals.

## Figures and Tables

**Table 1:** Comparative of the doses reported in animal models.

Species	Route Administrated	Dose	Reference
Adults			
Wistar rats	Caffeine (oral/single, bolus)	0.5, 15, or 45 mg/kg	[74]
Sprague Dawley rats	Instant coffee extract (oral/ single dose)	250 or 500 mg/ kg	[75]
Mongrel dogs	Caffeine (IV)	1, 3 and 5 mg/kg	[76]
Arabian horses	Caffeine (IV)	5 mg/kg	[77]
Neonates			
Lactating dairy cows	Caffeine (IV)	2 mg/kg	[78]
Rabbit New Zealand White	Caffeine (PO)	loading dose 20 mg/kg 10 mg/kg daily	[79]
Rabbits New Zealand White	Caffeine (IP)	10 mg/ kg daily	[47]
Wistar rats	Caffeine (IV)	10 mg/kg	[80]

IP: intraperitoneal; IV: intravenous; PO: oral.

## Data Availability

Not applicable.
